# You’d Better Zinc—Trace Element Homeostasis in Infection and Inflammation

**DOI:** 10.3390/nu11092078

**Published:** 2019-09-03

**Authors:** Hajo Haase, Lutz Schomburg

**Affiliations:** 1 Department of Food Chemistry and Toxicology, Berlin Institute of Technology, D-13355 Berlin, Germany; 2 TraceAge-DFG Research Unit on Interactions of Essential Trace Elements in Healthy and Diseased Elderly, Potsdam-Berlin-Jena D-13353, Germany; 3 Institut für Experimentelle Endokrinologie, Charité-Universitätsmedizin Berlin, Corporate Member of Freie Universität Berlin, Humboldt-Universität zu Berlin and Berlin Institute of Health, Südring 10, CVK, D-13353 Berlin, Germany

During recent years, we have witnessed a growing appreciation of several micronutrients in the immune response, including vitamins and minerals. Among them, the essential trace element zinc (Zn). Current knowledge on the role of Zn in infections and inflammation has recently been comprehensively reviewed by Nour Gammoh and Lothar Rink from RWTH Aachen University, Germany, as part of a special issue of Nutrients on Infectious and Inflammatory Diseases (https://www.mdpi.com/journal/nutrients/special_issues/nutrients_infectious_inflammatory_diseases). The authors highlight experimental evidence for the requirement of a sufficiently high and well-balanced Zn status, in order to allow the efficient functioning of both, the innate and adaptive immunity [1]. Unfortunately, in contrast with other trace elements, such as iron (Fe), selenium (Se) or copper (Cu), there are presently no established specific and reliable circulating biomarkers of the functional Zn status, which hampers straightforward clinical analyses from serum or plasma samples. Consequently, much clinical research relies on nutritional Zn intake assessment, and there is an emphasis on avoiding an insufficient Zn supply in certain populations at risk, such as children, seniors, women during pregnancy and lactation, or vegetarians [2]. While the current predominant marker of Zn status, i.e., total serum zinc, has several limitations, the potential relevance of free Zn concentrations has been suggested as a valuable readout of available Zn supply to target cells, as shown, e.g., in model systems of sepsis [3] or human serum [4]. Among the other accessible potential biomarkers of Zn supply are the metallothioneins in circulating leukocytes, that may likewise be associated with functional Zn status and Zn intake [5]. Many key processes of the immune system are affected by alterations in Zn availability, e.g., (i) the formation of extracellular traps by neutrophils, (ii) shifting the balance from humoral responses to immune cell-mediated immunity, and (iii) dynamically adapting the elevated inflammatory response by a better control of NF-κB activation to current needs, thereby avoiding overshooting immune cell activities [1]. Notably, the most stringent effects have been observed in models of Zn deficiency and moderate repletion. These insights are supported by respective clinical studies, collectively indicating that sub-optimal Zn status, due to low dietary intake, malfunction of the major Zn transport proteins of the ZIP (family SLC39) or ZnT (family SLC30) transporter type, are associated with impaired immune responses, diminished lymphocyte activities, and a number of immune-related diseases [6].

These pleiotropic consequences of Zn deficiency on the immune system, and thereby indirectly on a broad spectrum of disease risks, are reminiscent of another trace element implicated in redox regulation, antioxidative protection, and immune competence, i.e., the essential micronutrient Se. The first indications for an essential role of Zn and Se have been described almost contemporaneously around 60 years ago, when A.S. Prasad characterized clinical symptoms of Zn deficiency in malnourished subjects [7], and K. Schwarz and C.M. Foltz identified the essential role of Se in a rodent model of liver necrosis [8], respectively. While, both elements show a range of similarities with respect to their physiological role, disease regulation, and elevated health risks in relation to deficiency, there are also notable differences in their mode of action, relation to the immune system, and our understanding of their biology (Table 1).

It is estimated that about 10% of all human genes encode proteins, comprising known Zn-binding motifs, including the large group of Zn-finger-containing transcription factors, many catalytically active enzymes, and a number of other structural, binding or transporting proteins [29]. This variety of Zn-binding molecules contrasts with the small group of Se-dependent proteins that contain the essential trace element as the co-translationally inserted amino acid selenocysteine in their primary sequence, and are encoded in humans by a set of 25 genes only [30]. Refined mouse models and a small number of instructive inherited diseases are highlighting the relevance of particular selenoproteins for specific metabolic pathways, developmental aspects, and especially the regular functioning of the immune system [9,31]. The excessively larger Zn-dependent proteome hampers such unambiguous allocations of impaired cellular responses to alterations in Zn-status and to a particular Zn-dependent enzymatic activity, but the clear effects of Zn deficiency on immune cell activities underline the eminent role of a sufficiently high Zn status in health and disease [1,7,32]. Another fundamental difference between the roles of Se and Zn in infection and inflammation relates to the role of free Zn^2+^ ions, which have been described as potent intracellular and paracrine messengers, associated with tightly regulated machinery for intracellular storage, transient release, flow, reception, and effector functions [33]. Zn status directly controls immune cell activity by inhibiting several dephosphorylating enzymes, such as protein tyrosine phosphatases, acting as a second messenger in immune cells. It regulates signaling pathways originating from a variety of receptors for different mediators of infection, ranging from cytokines to pathogen-associated molecular patterns [6]. Similar activities are not described for Se, where all the major physiological and clinical effects are elicited via the selenocysteine-containing selenoproteins, as impressively underlined by the clinical characterization of the paradigmatic patients with inborn errors of selenoprotein biosynthesis and functions [34].

In general, there are many trace elements that are strongly interrelated with the immune system and vice versa, i.e., several cytokines affect the homeostasis of certain trace elements and conversely, certain trace elements control immune cell activity and the inflammatory response [35]. Serum Fe is a paradigmatic example, as it becomes regulated intensively during infection and inflammation as a negative acute phase reactant, potentially leading to anaemia of inflammation [36]. The down-regulation of circulating Fe serves mainly two purposes; pathogenic bacteria in blood become deprived of the essential trace element needed for their metabolism, thereby reducing proliferation and spreading (a process known as nutritional immunity), and host-specific microbicidal activities of lymphocytes that depend on a balanced Fe concentration are efficiently supported at the same time [37]. In the case of Fe, the molecular pathway mainly responsible for the fast and efficient alterations was partly disclosed with the identification of the liver-derived hormone hepcidin as the major regulator of Fe metabolism during an inflammatory response [38]. Besides Fe, Zn, and Se, an intense competition between some pathogens and the host is also observed for other less-well characterized trace elements, such as manganese (Mn) [39]. The use of trace elements as tools for limiting the viability of pathogens is not restricted to the extracellular space, but continues even after phagocytosis. Elegant studies have highlighted that intraphagosomal pathogens in macrophages are subject to killing by altered concentrations of trace metals. On one hand, Fe deprivation by natural resistance-associated membrane protein (NRAMP) 1 or Zn by sequestration to metallothioneins and into the Golgi apparatus occurs, while on the other hand, macrophages poison some phagocytosed bacteria, such as *Mycobacterium tuberculosis*, by elevating the concentrations of Zn and Cu within phagosomes to bactericidal amounts [40,41]. Next to their roles in nutritional immunity, Zn and Mn directly affect immune cell activities by partly converging pathways [42]. Recently, Mn was shown to increase the sensitivity of DNA sensor receptors during viral infection, thereby improving the anti-viral host defense [43]. Similarly, the Zn-dependent metallopeptidase STE24 (ZMPSTE24) acts as an important inhibitor of viral entry via its interaction with interferon-induced defense proteins with potential relevance for viral diseases, e.g., influenza, Zika, or Ebola infections [44].

It is long established that the concentration of yet another circulating trace element is intensively controlled under inflammatory conditions, namely copper (Cu), which belongs to the positive acute phase reactants [45]. Again, a complex network of Cu-dependent and Cu-regulating factors interacts to ensure a balanced Cu flow and to maximize its microbicidal effects [46]. Similar to serum Zn, Se, and Fe, the liver is once again the responsible tissue, affecting serum Cu concentrations decisively via cytokine-mediated regulation of ceruloplasmin biosynthesis and secretion into the circulation [47]. As Cu-loaded ceruloplasmin also acts as a potent ferroxidase affecting Fe redox state and intestinal resorption, a tight Fe-Cu interplay in gut and liver evolved, with high relevance for infection and inflammation [48].

In view of the essential importance of various trace elements in many different aspects of infection and inflammation, which are becoming increasingly recognized and their interactions understood, better dietary control and clinical monitoring of the trace element concentrations as health-promoting micronutrients and disease risk factors, as well as modifiers of convalescence and adjuvant therapeutics, is warranted. The synergistic interactions of trace elements, especially with respect to the immune system as a central and common endpoint of action, are of paramount importance for resolving inflammatory conditions and eliminating pathogenic invaders (Figure 1). Adequately powered clinical analyses and intervention trials are needed next for elucidating the role of health-relevant trace element deficiencies or inadequate nutritional or supplemental supply. This would allow an optimized individual support of a diseased subject in the clinics and a maximal protection of a healthy subject in the preventive setting. As such monitoring and adequate supportive interventions are cost-effective and safe, there is no reason for infected patients not to be receiving their supply of essential trace elements, as long as the recommended dosages are not surpassed and a relevant deficiency has been diagnosed. In clinics, as well as in ambulant medical care, trace element and micronutrient deficiencies are common findings, particularly in diseases with chronic inflammation [49] or infection [50]. It is high time to more seriously consider these unnecessary deficits, as they interfere with convalescence, worsen clinical presentation and disease course, reduce quality of life, are costly to the health care system, and add an unnecessary burden to the diseased organism in need of supportive medical and nutritional care.

## Figures and Tables

**Figure 1:**
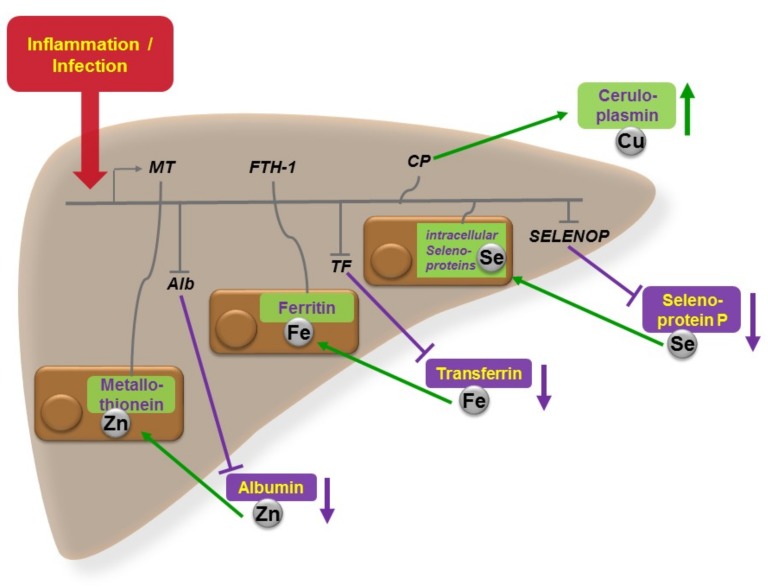
Central role of liver in acute phase regulation of circulating trace elements. In response to infection or inflammation, and the associated increase in pro-inflammatory cytokines, hepatocytes modify trace element metabolism, in order to deprive invading pathogens from supply with essential growth factors, and at the same time, improving the host’s anti-inflammatory response. The figure depicts schematically the effects on the distribution of copper (Cu), iron (Fe), selenium (Se), and zinc (Zn) between blood and liver. Serum Cu levels increase in response to inflammation mainly via up-regulated ceruloplasmin (CP) biosynthesis and secretion into blood. The hepatic hormone hepcidin diminishes Fe supply to the circulation and at the same time, transferrin concentrations decline, while intrahepatic ferritin (FTH-1) increases, collectively reducing circulating Fe levels (anaemia of inflammation). Hepatic selenoprotein biosynthesis becomes redirected in favor of certain intracellular selenoproteins and at the expense of the circulating Se-transport protein selenoprotein P (SELENOP). The re-distribution of Zn is more complex, involving all major organs, including immune cells and the gastrointestinal system, and a large number of Zn-dependent proteins [1]. A significant flow of Zn levels takes place from circulating albumin into hepatocytes and toward intracellular metallothioneins (MT).

**Table 1:** Parallels and differences in the interaction of Zn and Se with infection and inflammation.

	Zinc	(Reference)	Selenium	(Reference)
Effects of deficiency:				
infection rate	increased	[1]	increased	[9]
morbidity/mortality	increased	[1]	increased	[10,11]
inflammatory response	ambiguous	[1]	inadequate	[9,12]
NET formation	impaired	[1]	unknown	--
NF-κB activity	ambiguous	[1]	elevated	[9,13]
IL-6 concentrations	ambiguous	[1,14]	elevated	[9,14]
immune cell migration	impaired	[1]	impaired	[9,15]
oxidative stress/damage	elevated	[1,16]	elevated	[9,16]
lympho- to myelopoesis	shifted	[17]	unknown	--
Response to infection and inflammation:				
serum concentrations	decreased	[1,18]	decreased	[9,19]
hepatic concentrations	increased	[1,18]	ambiguous	[9,12]
Effects of supplementation on:				
mortality in sepsis	ambiguous	[20,21]	ambiguous	[22,23]
convalescence in sepsis	positive	[24]	positive	[23,25]
adverse effects in sepsis	none	[26,27]	none	[22,28]
